# Dietary Sodium Humate Improves Intestinal Mucosal Immune and Biological Barriers of Genetically Improved Farmed Tilapia (*Oreochromis niloticus*)

**DOI:** 10.1155/anu/3398904

**Published:** 2025-10-09

**Authors:** Bocheng Huang, Shuang Zhang, Xiaohui Dong, Shuyan Chi, Qihui Yang, Hongyu Liu, Shiwei Xie, Wei Zhang, Beiping Tan, Lei Guo, Changqing Yu, Junming Deng

**Affiliations:** ^1^College of Fisheries, Guangdong Ocean University, Zhanjiang 524088, China; ^2^Beijing Sloan Biological Technology Co., Ltd., Beijing 102206, China

**Keywords:** GIFT, intestinal microbiota, intestinal mucosa, intestinal mucosal immunological barrier, sodium humate

## Abstract

The present research examined the impact of sodium humate (SH) in feed on the intestinal health of genetically improved farmed tilapia (GIFT, *Oreochromis niloticus*). GIFT with average weight of 3.9 g were provided with diets incorporated diverse content of SH from low to high (0%, 0.1%, 0.2%, 0.4%, and 0.6%, denoted as SH0, SH1, SH2, SH4, and SH6) for 63 days. Results indicated that incorporating 0.4%–0.6% SH into the diet significantly reduced the plasma lactulose/rhamnose ratio, while plasma diamine oxidase (DAO) activity was depressed by supplementation with 0.1%–0.4% SH (*p* < 0.05). Dietary SH level increased the length and width of villu, as well as the levels of polymeric immunoglobulin receptor in the distal intestine (DI). The SH2 group exhibited elevated levels of interleukin (IL)-10 in the DI and transforming growth factor β1 (TGF-β1) and IL-1β in the proximal intestine (PI). On the other hand, the contents of tumor necrosis factor-α (TNF-α) in the middle intestine (MI), IL-1β, and IL-6 in the MI and the PI were generally lower in the SH groups contrast to the SH0 group, while interferon-gamma (IFN-γ) and IL-8 levels in the DI showed the opposite trend. Additionally, dietary inclusion of 0.2% SH promoted the intestinal microbiota species diversity and relative abundance of *Cetobacterium*, with SH6 group displaying the highest complexity. Overall, dietary SH supplementation improved intestinal health of GIFT by decreasing mucosal permeability, improving intestinal absorption surface area, enhancing intestinal immune barrier, and reduce inflammation. The recommended level of SH was 0.2%–0.4% of diet for GIFT.

## 1. Introduction

*Oreochromis niloticus*, ranked as the third most commonly cultivated fish globally, has achieved a production level of 4.5 million tons, representing 7.9% of total finfish production in 2022 [1]. Due to their rapid growth, disease resistance, adaptability to various production systems, flexible omnivorous diet, and ability to thrive at a low trophic level, Nile tilapia is extensively cultivated in intensive aquaculture [2]. Nevertheless, this farming practice presents challenges. To boost yields, aquaculture farmers increase stocking densities in limited spaces, causing overcrowding and water quality deterioration, making tilapia more susceptible to disease outbreaks [3, 4]. Traditionally, drugs or antibiotics have been employed to control disease incidence, but this strategy can result in drug resistance in fish or the presence of harmful residues that jeopardize food safety [5]. Consequently, researchers have been investigating alternative methods to enhance the health and immunity of aquaculture fish against infections, such as the formulation of dietary additives [6].

Humic acid (HA) is an organic compound from humic substances, formed through the long-term microbial decomposition of plant or animal matter. It contains multiple biologically active functional groups, including phenol, hydroxyl, carboxyl, and quinone [7]. HA is commonly present in soils and coal mines and can also be artificially synthesized [8]. These characteristics make HA an easily obtained and beneficial additive. Extensive research has explored on incorporating HA into the diets of poultry and livestock over the last 20 years [9, 10]. Recent academic attention on intestinal microbiota has underscored the potential of natural humic substances to enhance probiotics and inhibit pathogenic microorganisms in terrestrial animals [11, 12]. Increasing the abundance of beneficial intestinal flora through HA supplementation can improve the weight gain of weaned piglets [13]. In vitro experiments have additionally shown that humic substances significantly increase the protein synthesis by microbiota in the gastrointestinal tract of ruminants, leading to enhanced feed utilization and reduced methane emissions [14].

Research on the effects of HA in aquatic animals is limited compared to terrestrial vertebrates, but has shown positive outcomes. For example, a study on *Cyprinus carpio* found a significant improvement in growth with the addition of HA [15], similar results were seen in Asian seabass (*Lates calcarifer*) [16]. Additionally, incorporating 1.5% fulvic acid, a low molecular weight organic acid, into the diet improved intestinal health and promoted weight gain in loach (*Paramisgurnus dabryanus*) [12]. Similarly, supplementing 0.4% shilajit (a mineral rich in HA) into the diet significantly enhanced immunocompetence and growth of *Oreochromis mossambicus* [17]. Other studies have demonstrated that adding HA or its analogs to fish feeds can enhance digestive enzyme activities, antibacterial properties, and aid in the removal of lead from the fish's system [18, 19]. However, these studies have varied in direction, limited exploration of its impact on the intestines, an important organ. Therefore, further investigate the effects of HA, particularly its impact on the intestines is needed.

In preliminary study [20], we used sodium humate (SH) as a dietary additive in the feed of genetically improved farmed tilapia (GIFT)-strain tilapia, which significantly improved some nonspecific immunocompetence indices and digestibility indices, consequently boosting resistance against *Aeromonas hydrophila* and growth indices. This study aims to further previous research by comprehensively assessing the impact of various levels of dietary SH to the GIFT tilapia intestine, especially especially effects on intestinal mucosal immune, histomorphology, and biological barriers.

## 2. Materials and Methods

### 2.1. Experimental Diets

Five experimental diets were configured in accordance with the description of a previous study [20]. In brief, the control diet (SH0) was prepared with fish meal and common plant-based ingredients as protein sources achieving a crude protein level of 29%; the remaining feeds (SH1, 2, 4, and 6) were formulated to contain 1, 2, 4, and 6 g/kg SH (Beijing Sloan Biotechnology Co., Ltd., Beijing, China) in diet based on the SH group, respectively. The formulation of the experimental diets are listed in Table S1.

Protein ingredients and SH were individually ground into powder capable of passing through a 60-mesh sieve, just like other powdery solid raw materials. Subsequently, they were blended in the correct proportions, evenly mixed with lipid ingredients and water, and then, pressed into pellets with a size of Number 1.5 using an experimental pellet feed mill. The pellets were dried at 20°C for 12 h and finally stored at ‒20°C until needed.

### 2.2. Fish and Experimental Conditions

GIFT tilapia were sourced from a local fish farm and adjusted to the test setup by being temporarily housed in 3 m^3^ fiberglass tanks for 2 weeks. Following acclimation, 600 healthy fish, averaging 3.9 g each, were divided into five groups: SH0, SH1, SH2, SH4, and SH6. Each group had three replicates, initially containing 40 fish per tank. The tanks, with a capacity of 400 L filled to 300 L, were equipped with filtration systems and provided with natural illumination. Each feeding session was conducted until no fish were observed competing for food on the surface, indicating apparent satiation. The water was maintained within the range of 29–30°C using a heating rod, and the dissolved oxygen level was >6.0 mg/L.

### 2.3. Sample Collection

At the end of the experiment, the fish were first fasted a 24-h fasting period before being anesthetized with eugenol at a concentration of 1:12,000. Plasma was obtained using an anticoagulant-treated syringe and placed into 1.5 mL anticoagulant tube. These samples were then refrigerated at 4°C for 4 h, centrifuged at 6000 r/min, and stored at –80°C until analysis. The intestines were immediately excised and divided into proximal intestine (PI), middle intestine (MI), and distal intestine (DI) [21]. Mucosa samples from each intestinal segments of six fish in each tank were obtained by scraping and then stored at –80°C. Furthermore, the DI from two fish in each tank were placed in 10% formaldehyde fixing fluid for the preparation of DI sections. In addition, the intestinal contents of three fish were obtained in a sterile environment, and then, stored at –80°C for subsequent DNA extraction of bacteria.

### 2.4. Intestinal Mucosal Barrier Function Indicators Analyses

Plasma rhamnose and lactulose contents were evaluated following the procedure outlined by Tanha et al. [22]. The levels of plasma D-lactic acid (D-LA) and the activities of diamine oxidase (DAO) were quantified using colorimetric and spectrophotometric methods, respectively, with detection kits (Jiancheng Bioengineering Institute, Nanjing, China) operating according to the manual. Transforming growth factor-β1 (TGF-β1), tumor necrosis factor-α (TNF-α), interferon-gamma (IFN-γ), secretory immunoglobulin T (sIgT), poly immunoglobulin receptor (pIgR), interleukin (IL)-1β, IL-6, IL-8, IL-10, and IL-12 in the PI, MI, and DI were quantified using enzyme-linked immunosorbent assay kits for fish (R&D Systems, Minneapolis, USA).

### 2.5. Intestinal Morphological Observation

After fixation for 36 h, the DI samples were dehydrated in ethanol and then, embedded in paraffin wax. Subsequently, the sections measuring 4–6 µm in thickness were cut using a Leica Model BioCut Microtome (Leica, Wetzlar, Germany) and stained with hematoxylin and eosin. Measurements were taken of the length and width of six random villi, as well as the thickness of six different parts of the muscularis, using ImageJ software (Ver. 1.8.0, National Institutes of Health, USA).

### 2.6. Microbiota Analysis

#### 2.6.1. Total DNA Extraction and Detection

Intestinal contents samples were first washed with phosphate buffer saline and centrifuged, after which the supernatant was obtained and centrifuged. The resulting precipitates were then washed again with phosphate-buffered saline before being transferred. Total DNA was extracted following the protocol of the stool DNA Kit (Tiangen Biochemical Technology Co., Ltd, Beijing, China). Subsequently, the extracted DNA was spectrophotometrically quantified to assess its quality.

#### 2.6.2. 16S rDNA Polymerase Chain Reaction (PCR) Amplification, Mixing, and Purification

Amplification of 16S rDNA genes from distinct regions (V4) was carried out using specific primers (515F–806R) with barcodes. PCR reactions were carried out in a 30 μL volume, consisting of 15 μL of High-Fidelity PCR Mix (New England Biolabs, Beverly, USA), 0.2 μM of two kinds of primers, and 10 ng of the previously obtained sample template DNA. Amplification was executed in line with the amplification protocol described by Ma et al. [23]. Afterward, the productions were mixed in equal density ratios, purified using the Gene JETTM Gel Extraction Kit (Thermo Scientific, Waltham, USA), and visualized by electrophoresis on a 2% agarose gel with 1× SYB green loading buffer.

#### 2.6.3. Library Preparation and Sequencing

Sequencing library was created using the Ion Plus Fragment Library Kit and precisely evaluated with the Qubit 2.0 Fluorometer, both from Thermo Scientific (Waltham, USA). In the next step, sequencing was performed on an Ion S5XL System, generating single-end reads.

#### 2.6.4. Data Analysis

Single-end reads were matched by the unique barcodes, then, the barcode and primer sequences were trimmed to shorten the reads. Quality filtering was applied to the raw reads under specific conditions to produce high-quality clean reads [24]. Subsequently, reads were compared to a reference database using the UCHIME tool [25], which were then eliminated [26]. Further analysis was conducted using the Uparse algorithm in the USEARCH platform (https://www.drive5.com/usearch), where sequences with ≥97% similarity were clustered into identical operational taxonomic units (OTUs). In respect to every OTU, a sequence was selected to serve as the basis for further labeling and expansion. These sequences were utilized to compute the alpha diversity index, and sparse curves were constructed based on the Chao1, unique OTUs, and Shannon index. Unweighted unifrac was computed using QIIME (Version 1.9, University of Colorado, USA). To further elucidate the differences between each group, biomarkers were identified through LEfSe analysis (linear discriminant analysis effect size) conducted using LEfSe Online Tools (http://huttenhower.sph.harvard.edu/lefse).

### 2.7. Statistical Analysis

The data were presented as means with standard error (means ± SEM). One-way analysis of variance (ANOVA) was conducted, followed by the LSD test and Duncan's multiple comparison test. Statistical significance was defined as *p* < 0.05. Statistical analyses were all performed using SPSS (Ver. 16.0, IBM, Armonk, USA).

## 3. Results

### 3.1. Intestinal Mucosal Barrier Function

With the increase of dietary SH inclusion level, the rhamnose level linearly increased, while the plasma lactulose level generally decreased (Table 1). Thus, the plasma lactulose/rhamnose ratio steadily decreased with the increase of SH level in diet, which was higher in the SH0–SH2 groups than in the SH4–SH6 groups (*p* < 0.05). The plasma DAO activity was lower in the SH1, SH2, and SH4 groups compared to the SH0 and SH6 groups.

### 3.2. Intestinal Histomorphology

The villus height of DI generally increased with the increase of dietary SH level, which was lower in the SH0 group as opposed to the SH6 group (*p* < 0.05; Table 2). A similar trend was found in the villus width of DI, which was lower in the SH0 and SH1 groups compared to the SH4 group.

### 3.3. Intestinal Mucosal Immune Barrier

The mucosal sIgT level in MI was less in the SH2 group as opposed to the other groups (*p* < 0.05; Table 3). The mucosal pIgR level in DI gradually increased with the increase of dietary SH inclusion level, which was higher in the SH6 group than that in the SH0 group. However, in terms of the PI sIgT and pIgR, the MI pIgR, the DI sIgT levels, no significant disparities were found among the different dietary treatments (*p* > 0.05).

### 3.4. Anti-Inflammatory Cytokines in Intestinal Mucosa

With the increase of dietary SH inclusion level, the levels of TGF-β1 in PI mucosa and IL-10 in DI mucosa initially increased and subsequently decreased (Table 4). The TGF-β1 level in PI mucosa was higher in the SH2 group as opposed to the SH4 and SH6 groups, and the L-10 level in MI mucosa was higher in the SH1 and SH2 groups compared to the SH0, SH4, and SH6 (*p* < 0.05). However, no significant differences were found in the PI mucosal IL-10, the MI mucosal IL-10 and TGF-β1, and the DI mucosal TGF-β1 levels among the dietary treatments.

### 3.5. Pro-Inflammatory Cytokines in Intestinal Mucosa

The PI mucosal IL-6 level generally depressed with the increase of dietary SH inclusion level, and it was at a reduced level in the SH4 and SH6 groups as opposed to the SH0 group (*p* < 0.05; Table 5). The lowest IFN-γ level in the PI mucosa was found in the SH1 group, while the lowest IL-1β level in the PI mucosa was observed in the SH4 group.

The MI mucosal TNF-α level generally depressed with the increase of dietary SH inclusion level, which was lower in the H6 group as opposed to the SH0, SH1, and SH2 groups (*p* < 0.05; Table 5). However, the MI mucosal IL-1β level initially decreased and then enhanced with the increase of dietary SH inclusion level, which was less in the SH2 group compared to the SH0 group. The MI mucosal IL-6 level was less in the SH2 and SH6 groups as opposed to the SH0, SH1, and SH4 groups. There were no significant differences in the MI mucosal IFN-γ, IL-8, and IL-10 levels among the dietary treatment (*p* > 0.05).

The DI mucosal IFN-γ level first enhanced and then decreased with the increase of SH inclusion level in diet, which was higher in the SH2 group as opposed to the SH0 group (*p* < 0.05; Table 5). However, the DI mucosal IL-8 level generally increased with the increase of dietary SH inclusion level, which was higher in the SH6 group as opposed to the SH0 group. There were no significant differences in the DI mucosal TNF-α, IL-1β, IL-6, and IL-12 levels among the dietary treatments (*p* > 0.05).

### 3.6. Intestinal Mucosal Biological Barrier

**Figure 1:**
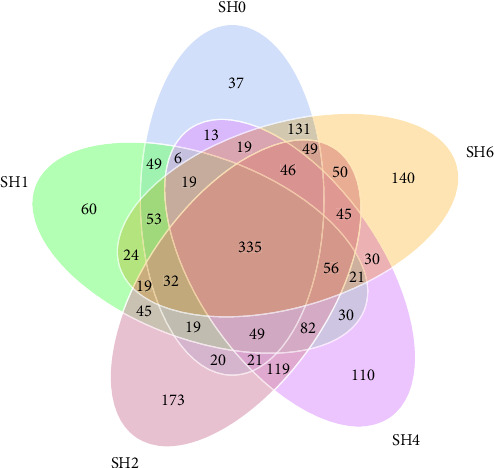
Common and unique operational taxonomic units (OTUs) of intestinal microbiota in tilapia with dietary various sodium humate levels. Each circle denotes a group, and the intersecting areas indicate the number of shared OTUs.

Figure 1 illustrates a Venn diagram displaying the common and OTUs across various groups, as identified through OTUs cluster analysis. Among all groups, there are 335 OTUs that are common. The SH2 group exhibited the greatest quantity of OTUs (173), whereas the SH0 group had the lowest count (37). The SH2 group demonstrated the highest values for observed species, Chao1 index, and ACE, while the lowest values for Shannon and Simpson indices were recorded in the SH0 and SH2 groups (Table 6).

**Figure 2:**
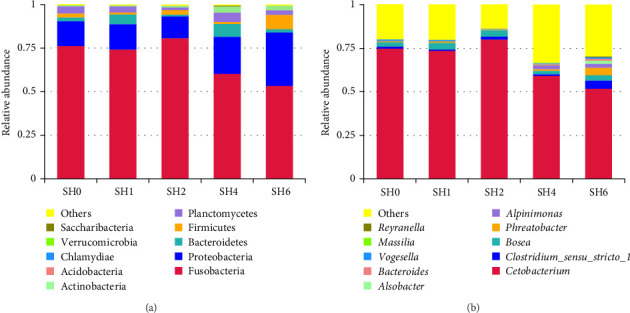
Effects of dietary various sodium humate levels on intestinal microbiota communities of tilapia. (A) Species level and (B) genus level.

Figure 2 illustrated the distribution of species on the phylum and genus tier. The top 10 microorganisms at the phylum level were Fusobacteria, Proteobacteria, Bacteroidetes, Firmicutes, Planctomycetes, Actinobacteria, Acidobacteria, Chlamydiae, Verrucomicrobia, and Saccharibacteria (Table 7). Fusobacteria emerged as the most abundant intestinal microbiota, with a significantly higher abundance in the SH2 group as opposed to the SH6 group. In contrast, Firmicutes exhibited a higher abundance in the SH6 group. The top 10 genera in relative abundance are *Cetobacterium*, *Clostridiumsensu_stricto_1*, *Bosea*, *Phreatobacter*, *Alpinimonas*, *Alsobacter*, *Bacteroides*, *Vogesella*, *Massilia*, and *Reyranella* (Table 8). *Cetobacterium* stand out as the predominant genus in the intestinal microbiota, showing a higher abundance in the SH2 group as opposed to the SH6 group. Conversely, the abundance of *Phreatobacter* is lower in the SH2 group compared to the SH6 group.

**Figure 3:**
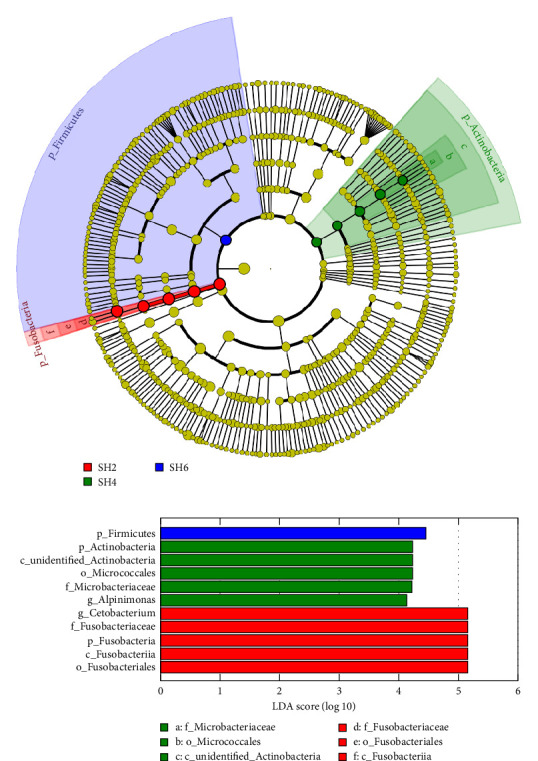
Differences in linear discriminant analysis effect size (LEfSe) values and evolutionary branches of intestinal microbiota in tilapia with dietary various sodium humate levels (SH0 vs. SH1 vs. SH2 vs. SH4 vs. SH6). The linear discriminant analysis (LDA) value distribution shows species with LDA score greater than 4, the biomarker with significantly difference between the groups. The bar chart represents the impact of the different species (LDA score). In the branching diagram of evolution, the circle of radiation represents the taxonomic rank from phylum to genus. At each classification level, every small circle represents a category within that level. The coloring principle: the differentially expressed biomarkers are colored with the group, each colored dot has an effect size LDA score.

Figure 3 depicted the comparison of LEfSe among the dietary treatments. In terms of phylum classification, the SH2, SH4, and SH6 groups exhibited microbial biomarkers including Fusobacteria, Actinobacteria, and Firmicutes. Additionally, on the genus tier, the SH2 and SH4 groups were associated with specific biomarkers, namely, *Cetobacterium* and *Alpinimonas*.

## 4. Discussion

Recent studies have shown that SH, when used as a dietary additive for fish or water quality improver, can positively impact growth performance, feed digestibility, and the health condition of fish. It also helps mitigate the adverse impacts of contaminants or pathogens [27–30]. For GIFT, our previous research has showed that incorporating 0.32% SH in diet significantly enhanced growth target and feed digestibility [20]. In this study, we further explored the effects of SH on the intestinal health of GIFT, highlighting its importance in nutrient absorption and as a barrier against pathogens and harmful antigens.

The absorption of nutrients by the intestine is influenced by its surface area, as indicated by the height and length of the intestinal villus in histomorphometry [31]. Previous studies have demonstrated that supplementing the diet with 0.25% humic material complex or 0.2% HA increased villus length and feed utilization [32, 33]. In this study, adding 0.2%–0.6% SH to GIFT diets yielded similar outcomes, indicating the beneficial effects of SH on intestinal growth and nutrient absorption.

The intestinal mucosa is crucial for defense mechanisms and proper intestinal function in fish [34]. Assessing intestinal barrier function involves evaluating mucosal permeability, as increased permeability can trigger inflammatory responses [35]. The enzyme DAO, found throughout the intestinal mucosa, typically has low plasma levels in healthy conditions, but significantly increases when mucosal integrity is compromised [36]. Thus, plasma DAO level can indicate the extent of intestinal mucosal permeability [37]. Additionally, the plasma levels of rhamnose and lactulose can serve as markers for assessing intestinal permeability [22]. It was demonstrated that increased intestinal permeability resulted in a decrease in the ability to absorb rhamnose compare to lactulose, leading to an elevated plasma lactulose/rhamnose ratio [38]. Thus, the current study's findings on plasma lactulose/rhamnose ratio and DAO activity suggest that adding of 0.1%–0.4% SH can enhance intestinal permeability of GIFT. Wang et al. [39] also demonstrated that SH can reduce serum DAO level in mice infected with *Salmonella Typhimurium*, by enhancing tight junction protein expression in intestine, supporting the results of the present study.

The intestinal mucosal immunological barrier is a key element of intestinal health of fish [40]. Secreted antibodies, such as teleost immunoglobulin T, are crucial for the intestinal mucosal immunity [41]. pIgR is essential for transporting immunoglobulins across epithelial cells to form slg in the mucosa [42]. With a relatively high abundance of quinone and phenolic functional groups endowing it with potent antioxidant capabilities, SH can effectively safeguard immune cells [43]. This study showed an enhance in pIgR content with dietary inclusion of SH, suggesting that SH may enhance the intestinal mucosal immunological barrier in GIFT. However, it should be noted that the addition of 0.2% SH reduced the content of sIgT in MI mucosa. This contrasts with studies on two terrestrial animals, laying hens and weaned calves, where the addition of HA to the feed increased serum IgA levels [44, 45]. Further research is needed to understand the reasons behind this discrepancy.

Inflammatory cytokines, which act as signaling molecules among immune cells, are essential to the inflammatory response and serve as indicators of the severity of intestinal inflammation in fish [46]. Among the numerous inflammatory factors, IL-10, TGF-β1, and others are considered anti-inflammatory cytokines [47]. This study revealed that in PI and DI, a low inclusion of SH (0.1%–0.2%) cause an increase in anti-inflammatory cytokines, while higher level of SH (0.6%) returned to baseline levels, suggesting that moderate addition of SH is beneficial in reducing intestinal inflammation, whereas high levels may have counterproductive effects. Sabi et al. [48] found that HA-like substances exhibit anti-inflammatory properties by reducing free radicals. In a study with rats, Ji et al. [49] demonstrated that SH accelerated wound contraction by upregulating TGF-β1 mRNA expression in skin and reducing the count of inflammatory cells during the early stages of injury. Comparable findings were observe in rats fed diets containing 80 mg/kg SH, where an increase of IL-10 content in serum led to reduced inflammation [50]. The suppression of pro-inflammatory cytokines may indicate a reduction in inflammation overreaction [51], while their upregulation could suggest an immunostimulatory properties [52]. According to the study in mice, SH can help attenuate intestinal inflammation induced by lipopolysaccharide [53] or enterotoxigenic *Escherichia coli* [54] by decreasing the intestinal levels of pro-inflammatory cytokine. In this study, slight variations in significant reductions of TNF-α and IL-1β contents in each intestinal segment were observed, all showing a significant decline within the 0.2%–0.6% SH addition range, indicating that SH possesses inflammation-reducing properties. It is worth noting that the IFN-γ level was significantly higher in the SH2 group than that in the SH0 group, and the IL-8 level showed an upward trend, which suggested that SH also has some immunostimulatory properties at the appropriate level of addition [55]. SH has been shown to form specialized glycoproteins in the body, binding to NK cells and T lymphocytes [56]. However, Wang et al. [39] confirmed that SH significantly decreases IFN-γ mRNA expression in colonic tissue and IFN-γ content in serum, thus, alleviating intestinal infection caused by *Salmonella Typhimurium*. This differs the findings of the current study, which possibly because of the lack of a negative control and the differences in experimental animals. In conclusion, as a preliminary experiment, given that the immune functions of different intestinal segments vary and the properties of SH may change with the pH within the intestinal segments, we examined each intestinal segment. These results indicate that the immune functions of all intestinal segments of GIFT have been affected by SH.

The intestinal mucosal biological barrier is the microbiota that in the intestine, and the diverse intestinal microbiota is extensively involved in the host's biological processes [57]. For fish, intestinal microbiota constitution is not only closely associated with the host's species characteristics but also to diet and environmental factors, such as geographical distribution, pH, or water temperature [58]. Previous studies identified that the dominant phylum in tilapia were Fusobacteria, Firmicides [59], Bacteroides, and Proteobacteria [60], as the dominant phyla in tilapia, aligning with the findings of this research. Additionally, Planctomycetes is a new major phylum found in the gut of GIFT in this study, contrasting with previous findings. Several studies indicate that in *Ctenopharyngodon idella*, ingestion of diets with appropriate carbohydrate-to-protein ratio or the supplementation of berberine, significantly enhanced the abundance of *Phreatobacter* in intestinal microbiota [61, 62]. Conversely, exposure to polyethylene microplastics decreased the relative abundance of *Phreatobacter* in zebrafish (*Danio rerio*) [63]. Although the impact of *Phreatobacter* and *Alpinimonas* on the host remains unexplored, in this study, they can be served as marker genera to evaluate the influence of a high SH diet on GIFT intestinal microbiota.

Certain genera within the Firmicutes, such as *Lactobacillus*, *Streptococcus*, *Leuconostoc*, and *Carnobacterium*, are prevalently acknowledged as probiotics and fulfill a vital function in the fish [47]. Studies on mice have indicated that SH can restore the decreased relative abundance of Firmicutes caused by the infection of *E. coli* [54]. Actinobacteria, another significant phylum, comprises short-chain fatty acid (SCFA)-producing bacteria that participate in dietary carbohydrate disintegration [64]. SCFAs can play multiple roles in the intestines, such as providing energy sources, modulating the equilibrium of intestinal microorganisms, and enhancing the function of the intestinal barrier [65]. Similarly, research has demonstrated a significant impact on the gut flora structure of laying hens, including a significant increase in the populations of Actinobacteria [66], which were accorded with the findings of the present study. Also in this study, *Cetobacterium* was identified as the predominant genus in the GIFT intestinal tract, consistent with previous research [59]. *Cetobacterium* is a gram-negative bacterium, most commonly found in the microbiota of freshwater fish [67]. According to the finding by Tsuchiya et al. [68], *Cetobacterium* in the intestines of fish can synthesize vitamin B12, which is then utilized by the host. Furthermore, recent research suggests that a strain of *Cetobacterium* sp. NK01, isolated from *O. niloticus*, synthesizes various amino acids and glycogen [69]. The abundance of *Cetobacterium* significantly increased with 0.2% SH supplementation in the present study, which indicate that at a certain level of SH addition can increase the abundance of some probiotics, thus, having a positive effect on the intestinal microbiota of GIFT. This may be associated with the biochemical mechanisms put forward in another study regarding how humic substances maintain bacterial activity, including membranotropic action (hydrophobicity), antioxidant activity, and potentially their ability to act as electron acceptors in the absence of oxygen [70].

## 5. Conclusion

Dietary 0.2%–0.4% SH inclusion has been shown to decrease the intestinal mucosal permeability, improve the boost immune barrier, reduce inflammation, and also modify the constitution of microbiota by spurring the growth of beneficial bacteria, ultimately enhancing the intestinal health of GIFT. While the addition of 0.6% SH further enhanced the level of anti-inflammatory factors and expand the intestinal absorption surface area, it did not improve the specific immunity and intestinal mucosal biological barrier as effectively as the addition of 0.2%–0.4%. This suggests that the biological properties of SH, such as anti-stress and immunostimulatory properties need to be added within a certain range to exert more pronounced effects. Therefore, the addition of 0.2%–0.4% SH can be considered beneficial as a dietary supplement in tilapia feed.

## Figures and Tables

**Table 1:** Effects of dietary various sodium humate levels on the intestinal mucosal barrier function of tilapia.

Item	SH0	SH1	SH2	SH4	SH6
Lactulose (pg/L)	0.58 ± 0.01^c^	0.58 ± 0.02^c^	0.63 ± 0.02^c^	0.49 ± 0.01^b^	0.41 ± 0.01^a^
Rhamnose (pg/L)	1.33 ± 0.05^a^	1.47 ± 0.04^a,b^	1.50 ± 0.03^a,b^	1.52 ± 0.05^a,b^	1.63 ± 0.11^b^
Lactulose/rhamnose	0.43 ± 0.02^c^	0.39 ± 0.02^c^	0.42 ± 0.02^c^	0.32 ± 0.02^b^	0.25 ± 0.01^a^
D-lactic acid (ng/L)	0.23 ± 0.00	0.24 ± 0.01	0.24 ± 0.05	0.24 ± 0.01	0.22 ± 0.00
Diamine oxidase (U/L)	23.40 ± 1.65^b^	12.46 ± 1.99^a^	12.34 ± 1.63^a^	12.57 ± 0.53^a^	27.59 ± 0.88^b^

*Note:* Values are means ± SEM (*n* = 3). Different superscript letters in the same row indicate significant differences (*p* < 0.05).

**Table 2:** Effects of dietary various sodium humate levels on the distal intestinal morphology of tilapia.

Item	SH0	SH1	SH2	SH4	SH6
Villus height (μm)	175.00 ± 4.16^a^	181.12 ± 4.33^a,b^	190.44 ± 4.67^a,b^	187.72 ± 5.90^a,b^	191.16 ± 5.97^b^
Villus width (μm)	95.42 ± 3.78^a^	94.20 ± 2.31^a^	97.44 ± 2.59^a,b^	105.79 ± 3.47^b^	100.06 ± 3.40^a,b^
Muscularis thickness (μm)	51.22 ± 1.70	52.24 ± 1.37	55.30 ± 2.16	52.47 ± 1.68	54.81 ± 1.94

*Note:* Values are means ± SEM (*n* = 18). Different superscript letters in the same row indicate significant differences (*p* < 0.05).

**Table 3:** Effects of dietary various sodium humate levels on the intestinal mucosal immune barrier of tilapia.

Item	SH0	SH1	SH2	SH4	SH6
Proximal intestine					
sIgT (mg/g protein)	0.28 ± 0.00	0.26 ± 0.02	0.34 ± 0.01	0.33 ± 0.05	0.32 ± 0.04
pIgR (pg/g protein)	0.16 ± 0.01	0.15 ± 0.01	0.17 ± 0.01	0.14 ± 0.02	0.17 ± 0.02
Middle intestine					
sIgT (mg/g protein)	0.52 ± 0.03^b^	0.54 ± 0.01^b^	0.37 ± 0.04^a^	0.55 ± 0.01^b^	0.57 ± 0.07^b^
pIgR (pg/g protein)	0.15 ± 0.01	0.18 ± 0.02	0.16 ± 0.00	0.17 ± 0.00	0.19 ± 0.04
Distal intestine					
sIgT (mg/g protein)	0.54 ± 0.08	0.62 ± 0.05	0.51 ± 0.11	0.59 ± 0.13	0.57 ± 0.03
pIgR (pg/g protein)	0.18 ± 0.03^a^	0.24 ± 0.04^a,b^	0.27 ± 0.04^a,b^	0.26 ± 0.01^a,b^	0.30 ± 0.03^b^

*Note:* Values are means ± SEM (*n* = 3). Different superscript letters in the same row indicate significant differences (*p* < 0.05).

Abbreviations: pIgR, polymeric immunoglobulin receptor; sIgT, secretory immunoglobulin T.

**Table 4:** Effects of dietary various sodium humate levels on anti-inflammatory cytokines in intestinal mucosa of tilapia.

Item	SH0	SH1	SH2	SH4	SH6
Proximal intestine					
IL-10 (pg/g protein)	0.20 ± 0.02	0.20 ± 0.02	0.24 ± 0.01	0.22 ± 0.02	0.21 ± 0.01
TGF-β1 (ng/g protein)	18.03 ± 0.71^a,b^	16.49 ± 0.96^a,b^	20.21 ± 1.51^b^	15.13 ± 1.59^a^	15.60 ± 0.57^a^
Middle intestine					
IL-10 (pg/g protein)	0.26 ± 0.00	0.29 ± 0.01	0.25 ± 0.01	0.30 ± 0.05	0.25 ± 0.03
TGF-β1 (ng/g protein)	17.69 ± 2.67	18.32 ± 2.32	15.24 ± 0.64	19.90 ± 1.54	17.54 ± 6.03
Distal intestine					
IL-10 (pg/g protein)	0.30 ± 0.04^a^	0.42 ± 0.01^b^	0.45 ± 0.05^b^	0.30 ± 0.05^a^	0.26 ± 0.01^a^
TGF-β1 (ng/g protein)	22.57 ± 0.82	25.42 ± 4.72	24.35 ± 3.58	21.51 ± 1.98	23.99 ± 1.42

*Note:* Values are means ± SEM (*n* = 3). Different superscript letters in the same row indicate significant differences (*p* < 0.05).

Abbreviations: IL-10, interleukin 10; TGF-β1, transforming growth factor β1.

**Table 5:** Effects of dietary various sodium humate levels on pro-inflammatory cytokines in intestinal mucosa of tilapia.

Item	SH0	SH1	SH2	SH4	SH6
Proximal intestine					
TNF-α (ng/g protein)	26.95 ± 1.28	24.04 ± 1.57	28.32 ± 0.43	23.45 ± 3.26	24.39 ± 0.96
IFN-γ (ng/g protein)	41.74 ± 1.98^a,b^	36.97 ± 0.69^a^	50.34 ± 4.29^b^	39.32 ± 3.77^a,b^	47.75 ± 4.42^a,b^
IL-1β (ng/g protein)	12.84 ± 0.43^c^	11.68 ± 0.29^b,c^	13.19 ± 0.43^c^	8.77 ± 1.00^a^	10.79 ± 0.30^b^
IL-6 (ng/g protein)	40.22 ± 1.78^b^	30.81 ± 5.90^a,b^	30.73 ± 0.91^a,b^	26.70 ± 1.85^a^	29.41 ± 1.17^a^
IL-8 (ng/g protein)	6.64 ± 0.67	5.68 ± 0.61	7.19 ± 0.43	6.54 ± 1.00	7.17 ± 0.91
IL-12 (ng/g protein)	2.32 ± 0.12	2.03 ± 0.11	2.50 ± 0.01	2.56 ± 0.41	2.34 ± 0.27
Middle intestine					
TNF-α (ng/g protein)	31.55 ± 4.86^b^	30.76 ± 3.94^b^	30.79 ± 0.77^b^	28.98 ± 1.28^a,b^	19.88 ± 1.18^a^
IFN-γ (ng/g protein)	38.27 ± 2.04	44.54 ± 6.61	43.07 ± 0.02	45.59 ± 1.81	44.58 ± 13.55
IL-1β (ng/g protein)	14.86 ± 0.93^b^	11.60 ± 1.62^a,b^	10.11 ± 0.28^a^	11.25 ± 0.36^a,b^	10.90 ± 2.18^a,b^
IL-6 (ng/g protein)	40.67 ± 3.20^b^	41.85 ± 0.49^b^	28.45 ± 1.19^a^	42.43 ± 0.64^b^	33.36 ± 1.73^a^
IL-8 (ng/g protein)	6.26 ± 0.99	6.41 ± 0.91	5.99 ± 0.80	5.46 ± 0.13	5.50 ± 1.69
IL-12 (ng/g protein)	2.44 ± 0.43	2.40 ± 0.37	2.68 ± 0.33	2.47 ± 0.15	2.29 ± 0.68
Distal intestine					
TNF-α (ng/g protein)	32.78 ± 2.07	48.84 ± 0.42	50.04 ± 8.41	47.24 ± 6.03	48.06 ± 5.20
IFN-γ (ng/g protein)	49.07 ± 7.25^a^	63.45 ± 3.51^a,b^	77.85 ± 12.42^b^	64.64 ± 7.11^a,b^	70.28 ± 5.73^a,b^
IL-1β (ng/g protein)	12.49 ± 1.23	14.53 ± 1.97	18.53 ± 2.45	18.80 ± 2.88	18.19 ± 1.09
IL-6 (ng/g protein)	42.75 ± 1.90	61.40 ± 0.26	58.40 ± 12.46	44.58 ± 9.55	43.56 ± 3.55
IL-8 (ng/g protein)	8.42 ± 0.37^a^	10.74 ± 1.77^a,b^	10.33 ± 1.21^a,b^	9.52 ± 0.29^a,b^	12.51 ± 0.72^b^
IL-12 (ng/g protein)	3.05 ± 0.25	3.99 ± 0.78	3.46 ± 0.19	3.05 ± 0.30	4.00 ± 0.36

*Note:* Values are means ± SEM (*n* = 3). Different superscript letters in the same row indicate significant differences (*p* < 0.05).

Abbreviations: IFN-γ, interferon γ; IL-12, interleukin 12; IL-1β, interleukin 1β; IL-6, interleukin 6; IL-8, interleukin 8; TNF-α, tumor necrosis factor α.

**Table 6:** Effects of dietary various sodium humate levels on the microbiota alpha diversity of tilapia.

Item	SH0	SH1	SH2	SH4	SH6
Observed_species	326.33 ± 31.41^a^	416.33 ± 16.84^a,b^	454.17 ± 45.95^b^	385.33 ± 39.23^a,b^	381.00 ± 40.08^a,b^
Shannon	1.63 ± 0.28^a^	1.69 ± 0.15^a^	1.66 ± 0.16^a^	2.21 ± 0.19^a,b^	2.74 ± 0.26^b^
Simpson	0.38 ± 0.09^a^	0.43 ± 0.05^a,b^	0.35 ± 0.05^a^	0.58 ± 0.05^b,c^	0.67 ± 0.06^c^
Chao1	387.34 ± 36.96^a^	480.04 ± 18.59^a,b^	522.02 ± 52.71^b^	444.88 ± 45.85^a,b^	439.04 ± 42.97^a,b^
ACE	400.41 ± 37.20^a^	496.37 ± 18.96^a,b^	539.78 ± 51.23^b^	455.71 ± 45.28^a,b^	449.30 ± 44.12^a,b^

*Note:* Values are means ± SEM (*n* = 6). Different superscript letters in the same row indicate significant differences (*p* < 0.05). Shannon index: the index of the total number of categories in the sample and the proportion of each category; Chao1 index: a measure of OTUs in a sample; ACE: estimate the actual number of species present in the community.

Abbreviation: ACE, abundance-based coverage estimator.

**Table 7:** Effects of various sodium humate levels on the structure and abundance of intestinal microbiota in tilapia (phylum level).

Item	SH0	SH1	SH2	SH4	SH6
Fusobacteria	76.3869 ± 8.5965^a,b^	74.3806 ± 4.1920^a,b^	80.9347 ± 3.3422^b^	60.3520 ± 5.3635^a,b^	53.6530 ± 7.3484^a^
Proteobacteria	14.1337 ± 5.6342	14.5263 ± 3.5637	12.4087 ± 3.3274	21.3457 ± 4.3484	30.3016 ± 9.1274
Bacteroidetes	2.2773 ± 1.6853	5.6996 ± 3.9028	1.0016 ± 0.6054	7.4519 ± 4.0795	2.0634 ± 1.0110
Firmicutes	2.1825 ± 0.4194^a^	1.2517 ± 0.2115^a^	2.8243 ± 0.8196^a^	1.0673 ± 0.3740^a^	8.4346 ± 3.8591^b^
Planctomycetes	4.2865 ± 2.5757	3.5112 ± 1.7702	1.4616 ± 0.5182	5.7020 ± 3.0265	2.3847 ± 0.8404
Actinobacteria	0.3564 ± 0.0980	0.4076 ± 0.1365	0.9852 ± 0.1803	3.6049 ± 1.3055	2.8145 ± 1.5740
Acidobacteria	0.0042 ± 0.0016	0.0146 ± 0.0048	0.0513 ± 0.0322	0.1125 ± 0.1045	0.0038 ± 0.0013
Chlamydiae	0.1118 ± 0.0882	0.0073 ± 0.0002	0.0138 ± 0.0045	0.0120 ± 0.0036	0.0200 ± 0.0081
Verrucomicrobia	0.1320 ± 0.0594	0.0848 ± 0.0499	0.1349 ± 0.0408	0.1240 ± 0.0379	0.0621 ± 0.0252
Saccharibacteria	0.0009 ± 0.0007	0.0051 ± 0.0029	0.0513 ± 0.0399	0.0224 ± 0.0086	0.0022 ± 0.0015
Others	0.1276 ± 0.0308	0.1112 ± 0.0334	0.1327 ± 0.0264	0.2053 ± 0.0570	0.2601 ± 0.1091

*Note:* Values are means ± SEM (*n* = 6). Different superscript letters in the same row indicate significant differences (*p* < 0.05).

**Table 8:** Effects of various sodium humate levels on the structure and abundance of intestinal microbiota in tilapia (genus level).

Item	SH0	SH1	SH2	SH4	SH6
*Cetobacterium*	74.9153 ± 8.4145^a,b^	73.8662 ± 4.1577^a,b^	80.1234 ± 3.2403^b^	59.4001 ± 5.2189^a,b^	51.8484 ± 7.0923^a^
*Clostridium_sensu_stricto_1*	1.0090 ± 0.4541	0.6408 ± 0.1214	1.9027 ± 0.7617	0.6204 ± 0.2982	4.8834 ± 2.5134
*Bosea*	2.5666 ± 1.1142	3.6659 ± 2.0869	3.4891 ± 1.8297	2.3636 ± 0.9885	3.0710 ± 1.0879
*Phreatobacter*	0.4689 ± 0.1498^a^	0.9797 ± 0.6127^a,b^	0.1897 ± 0.0165^a^	0.7101 ± 0.3748^a,b^	4.3635 ± 1.9510^b^
*Alpinimonas*	0.0970 ± 0.0468	0.1003 ± 0.0389	0.2124 ± 0.0502	2.6698 ± 1.0857	2.3095 ± 1.5665
*Alsobacter*	2.5793 ± 1.1187	3.6948 ± 2.1043	3.6169 ± 1.8041	2.3922 ± 0.9831	3.0868 ± 1.0945
*Bacteroides*	0.1085 ± 0.0897	0.0133 ± 0.0050	0.0095 ± 0.0033	0.0708 ± 0.0586	0.8960 ± 0.7451
*Vogesella*	0.9286 ± 0.2545	0.6783 ± 0.2802	0.3621 ± 0.1136	0.4245 ± 0.1209	0.9413 ± 0.4669
*Massilia*	0.0011 ± 0.0005	0.0162 ± 0.0157	0.0264 ± 0.0121	0.4462 ± 0.4393	0.0020 ± 0.0011
*Reyranella*	0.1380 ± 0.0705	0.0202 ± 0.0030	0.0448 ± 0.0175	0.0275 ± 0.0010	0.5148 ± 0.3940
Others	19.7119 ± 6.9768^a,b^	20.0179 ± 4.0996^a,b^	13.6281 ± 1.4743^a^	33.2650 ± 4.2613^b^	29.5696 ± 4.6758^a,b^

*Note:* Values are means ± SEM (*n* = 6). Different superscript letters in the same row indicate significant differences (*p* < 0.05).

## Data Availability

The data that support the findings of this study are available upon request from the corresponding author.

## Supporting Information

Supporting Information Due to space constraints, formulation and proximate composition of the experimental diets (Table S1) are included in the Supporting Information.

## References

[1] FAO (2022). *The State of World Fisheries and Aquaculture 2022*.

[2] Ramos M., Batista S., Pires M. (2017). Dietary Probiotic Supplementation Improves Growth and the Intestinal Morphology of Nile Tilapia. *Animal*.

[3] Tan H. Y., Chen S.-W., Hu S.-Y. (2019). Improvements in the Growth Performance, Immunity, Disease Resistance, and Gut Microbiota by the Probiotic Rummeliibacillus Stabekisii in Nile Tilapia (*Oreochromis niloticus*). *Fish & Shellfish Immunology*.

[4] Prokešová M. D., Gebauer T., Korytář T. (2024). Performance, Immune Response, Disease Resistance, and Gut Microbiota of Rainbow Trout, *Oncorhynchus mykiss* (Walbaum, 1792) Juveniles Fed Ground Leonardite With a High Humic Substance Content. *Aquaculture*.

[5] Liu X., Steele J. C., Meng X.-Z. (2017). Usage, Residue, and Human Health Risk of Antibiotics in Chinese Aquaculture: A Review. *Environmental Pollution*.

[6] Dawood M. A. O., Koshio S., Esteban M. Á. (2018). Beneficial Roles of Feed Additives as Immunostimulants in Aquaculture: A Review. *Reviews in Aquaculture*.

[7] Louvado A., Cleary D. F., Pereira L. F. (2021). Humic Substances Modulate Fish Bacterial Communities in a Marine Recirculating Aquaculture System. *Aquaculture*.

[8] Yang F., Zhang S., Cheng K., Antonietti M. (2019). A Hydrothermal Process to Turn Waste Biomass into Artificial Fulvic and Humic Acids for Soil Remediation. *Science of the Total Environment*.

[9] Arif M., Alagawany M., Abd El-Hack M. E., Saeed M., Arain M. A., Elnesr S. S. (2019). Humic Acid as a Feed Additive in Poultry Diets: A Review. *Iranian Journal of Veterinary Research*.

[10] Kholif A. E., Matloup O. H., EL-Bltagy E. A., Olafadehan O. A., Sallam S. M. A., El-Zaiat H. M. (2021). Humic Substances in the Diet of Lactating Cows Enhanced Feed Utilization, Altered Ruminal Fermentation, and Improved Milk Yield and Fatty Acid Profile. *Livestock Science*.

[11] Yegani M., Korver D. R. (2008). Factors Affecting Intestinal Health in Poultry. *Poultry Science*.

[12] Gao Y., He J., He Z. (2017). Effects of Fulvic Acid on Growth Performance and Intestinal Health of Juvenile Loach *Paramisgurnus dabryanus* (Sauvage). *Fish & Shellfish Immunology*.

[13] Visscher C., Hankel J., Nies A. (2019). Performance, Fermentation Characteristics and Composition of the Microbiome in the Digest of Piglets Kept on a Feed With Humic Acid-Rich Peat. *Frontiers in Veterinary Science*.

[14] Sheng P., Ribeiro G. O., Wang Y., McAllister T. A. (2019). Humic Substances Reduce Ruminal Methane Production and Increase the Efficiency of Microbial Protein Synthesis *in vitro*. *Journal of the Science of Food and Agriculture*.

[15] Wahab A. M. A., Refaee A. El, Ammar A. A. (2012). Effects of Humic Acid as Feed Additive in Improvement of Nonspecific Immune Response and Disease Resistance in Common Carp (*Cyprinus carpio*). *Egyptian Journal for Aquaculture*.

[16] Rasidi R., Jusadi D., Setiawati M., Yuhana M., Zairin M., Sugama K. (2021). Dietary Supplementation of Humic Acid in the Feed of Juvenile Asian Seabass, *Lates calcarifer* to Counteract Possible Negative Effects of Cadmium Accumulation on Growth and Fish Well-Being When Green Mussel (*Perna viridis*) Is Used as a Feed Ingredient. *Aquaculture Research*.

[17] Musthafa M. S., Asgari S. M., Elumalai P., Hoseinifar S. H., Van Doan H. (2018). Protective Efficacy of Shilajit Enriched Diet on Growth Performance and Immune Resistance Against *Aeromonas hydrophila* in *Oreochromis mossambicus*. *Fish & Shellfish Immunology*.

[18] Supriyono E., Nirmala K., Harris E., Affandi R., Jusadi D. (2017). Bio-Elimination of Lead (Pb) From the Organs of Red Tilapia (*Oreochromis* sp.) Using *Gliricidia sepium* Compost as a Feed Additive. *AACL Bioflux*.

[19] Yılmaz S., Ergun S., Çelik E. Ş., Yigit M. (2018). Effects of Dietary Humic Acid on Growth Performance, Haemato-Immunological and Physiological Responses and Resistance of Rainbow Trout, *Oncorhynchus mykiss* to *Yersinia ruckeri*. *Aquaculture Research*.

[20] Deng J., Lin B., Zhang X. (2020). Effects of Dietary Sodium Humate on Growth, Antioxidant Capacity, Non-Specific Immune Response, and Resistance to *Aeromonas hydrophila* in Genetic Improvement of Farmed Tilapia (GIFT, *Oreochromis niloticus*). *Aquaculture*.

[21] Pirarat N., Pinpimai K., Endo M. (2011). Modulation of Intestinal Morphology and Immunity in Nile Tilapia (*Oreochromis niloticus*) by, *Lactobacillus rhamnosus*, GG. *Research in Veterinary Science*.

[22] Tanha P. M., Øverland M., Landsverk T. (2016). Bacterial Translocation and in Vivo Assessment of Intestinal Barrier Permeability in Rainbow Trout (*Oncorhynchus mykiss*) With and Without Soyabean Meal-Induced Inflammation. *Journal of Nutritional Science*.

[23] Ma X., Bi Q., Kong Y. (2022). Dietary Lipid Levels Affected Antioxidative Status, Inflammation Response, Apoptosis and Microbial Community in the Intestine of Juvenile Turbot (*Scophthalmus maximus L*.). *Comparative Biochemistry and Physiology Part A: Molecular & Integrative Physiology*.

[24] Kechin A., Boyarskikh U., Kel A., Filipenko M. (2017). cutPrimers: A New Tool for Accurate Cutting of Primers From Reads of Targeted Next Generation Sequencing. *Journal of Computational Biology*.

[25] Edgar R. C., Haas B. J., Clemente J. C., Quince C., Knight R. (2011). UCHIME Improves Sensitivity and Speed of Chimera Detection. *Bioinformatics*.

[26] Haas B. J., Gevers D., Earl A. M. (2011). Chimeric 16S rRNA Sequence Formation and Detection in Sanger and 454-Pyrosequenced PCR Amplicons. *Genome Research*.

[27] Yamin G., Zilberg D., Levy G., van Rijn J. (2017). The Protective Effect of Humic-Rich Substances From Monogenean Parasites Infecting the Guppy (*Poecilia reticulata*). *Aquaculture*.

[28] Prokešová M., Bušová M., Zare M. (2021). Effect of Humic Substances as Feed Additive on the Growth Performance, Antioxidant Status, and Health Condition of African Catfish (*Clarias gariepinus*, Burchell 1822). *Animals*.

[29] Reham A. A., Mounes H. A. M., Ahmed K. M. (2019). Use of Humic Acid and Yucca Extract as a Benefactor on Water Quality and Their Impact on Some Hematological and Histological Parameters of *Oreochromis niloticus*. *Egyptian Journal of Aquatic Biology and Fisheries*.

[30] Lieke T., Steinberg C. E., Pan B. (2021). Phenol-Rich Fulvic Acid as a Water Additive Enhances Growth, Reduces Stress, and Stimulates the Immune System of Fish in Aquaculture. *Scientific Reports*.

[31] Geda F., Rekecki A., Decostere A. (2012). Changes in Intestinal Morphology and Amino Acid Catabolism in Common Carp at Mildly Elevated Temperature as Affected by Dietary Mannanoligosaccharides. *Animal Feed Science and Technology*.

[32] Taklimi S. M. S. M., Ghahri H., Isakan M. A. (2012). Influence of Different Levels of Humic Acid and Esterified Glucomannan on Growth Performance and Intestinal Morphology of Broiler Chickens. *Agricultural Sciences*.

[33] Mohammadsadeghi F., Afsharmanesh M., Ebrahimnejad H. (2019). The Substitution of Humic Material Complex With Mineral Premix in Diet and Interaction of that With Probiotic on Performance, Intestinal Morphology and Microflora of Chickens. *Livestock Science*.

[34] Liu Y., Fan J., Huang H. (2022). High Dietary Non-Starch Polysaccharides Detrimental to Nutrient Digestibility, Digestive Enzyme Activity, Growth Performance, and Intestinal Morphology in Largemouth Bass, *Micropterus salmoides*. *Frontiers in Nutrition*.

[35] Sittipo P., Choi J., Lee S., Lee Y. K. (2022). The Function of Gut Microbiota in Immune-Related Neurological Disorders: A Review. *Journal of Neuroinflammation*.

[36] Zhu R., Li L., Li M. (2021). Effects of Dietary Glycinin on the Growth Performance, Immunity, Hepatopancreas and Intestinal Health of Juvenile, *Rhynchocypris lagowskii*, Dybowski. *Aquaculture*.

[37] Liu X., Wu P., Jiang W.-D. (2021). Effects of Dietary Ochratoxin A on Growth Performance and Intestinal Apical Junctional Complex of Juvenile Grass Carp (*Ctenopharyngodon idella*). *Toxins*.

[38] Van Nieuwenhoven M. A., Geerling B. J., Deutz N. E. P., Brouns F., Brummer R.-J. M. (1999). The Sensitivity of the Lactulose/Rhamnose Gut Permeability Test. *European Journal of Clinical Investigation*.

[39] Wang D., Zheng Y., Fan Y. (2023). Sodium Humate-Derived Gut Microbiota Ameliorates Intestinal Dysfunction Induced by *Salmonella Typhimurium* in Mice. *Microbiology Spectrum*.

[40] Zhang H., Ran C., Teame T. (2020). Research Progress on Gut Health of Farmers Teleost Fish: A Viewpoint Concerning the Intestinal Mucosal Barrier and the Impact of Its Damage. *Reviews in Fish Biology and Fisheries*.

[41] Danilova N., Bussmann J., Jekosch K., Steiner L. A. (2005). The Immunoglobulin Heavy-Chain Locus in Zebrafish: Identification and Expression of a Previously Unknown Isotype, Immunoglobulin Z. *Nature Immunology*.

[42] Turula H., Wobus C. E. (2018). The Role of the Polymeric Immunoglobulin Receptor and Secretory Immunoglobulins During Mucosal Infection and Immunity. *Viruses*.

[43] Wang R., Wang Y., An X. (2023). Effects of Biomacromolecules on Growth, Digestibility, Digestive Enzyme Activity, Antioxidation, and Immunity in Broilers. *South African Journal of Animal Science*.

[44] Wang D., Du Y., Huang S., You Z., Zheng D., Liu Y. (2021). Combined Supplementation of Sodium Humate and Glutamine Reduced Diarrhea Incidence of Weaned Calves by Intestinal Microbiota and Metabolites Changes. *Journal of Animal Science*.

[45] Zhang A., Pirzado S., Liu G. (2020). Dietary Supplementation With Sodium Humate Improves Egg Quality and Immune Function of Laying Hens. *Journal of Applied Animal Nutrition*.

[46] Dong Y. W., Feng L., Jiang W. D. (2018). Dietary Threonine Deficiency Depressed the Disease Resistance, Immune and Physical Barriers in the Gills of Juvenile Grass Carp (*Ctenopharyngodon idella*) Under Infection of *Flavobacterium columnare*. *Fish & Shellfish Immunology*.

[47] Huang B., Zhang S., Dong X. (2022). Effects of Fishmeal Replacement by Black Soldier Fly on Growth Performance, Digestive Enzyme Activity, Intestine Morphology, Intestinal Flora and Immune Response of Pearl Gentian Grouper (*Epinephelus fuscoguttatus*♀× *Epinephelus lanceolatus*♂). *Fish & Shellfish Immunology*.

[48] Sabi R., Vrey P., van Rensburg C. E. J. (2012). Carbohydrate-Derived Fulvic Acid (CHD-FA) Inhibits Carrageenan-Induced Inflammation and Enhances Wound Healing: Efficacy and Toxicity Study in Rats. *Drug Development Research*.

[49] Ji Y., Zhang A., Chen X., Che X., Zhou K., Wang Z. (2016). Sodium Humate Accelerates Cutaneous Wound Healing by Activating TGF-β/Smads Signaling Pathway in Rats. *Acta Pharmaceutica Sinica B*.

[50] Çalışır M., Akpınar A., Poyraz Ö., Göze F., Çınar Z. (2016). The Histopathological and Morphometric Investigation of the Effects of Systemically Administered Humic Acid on Alveolar Bone Loss in Ligature-Induced Periodontitis in Rats. *Journal of Periodontal Research*.

[51] Fan Z., Wu D., Li J., Li C., Zheng X., Wang L. (2022). Phosphorus Nutrition in Songpu Mirror Carp (*Cyprinus carpio* Songpu) During Chronic Carbonate Alkalinity Stress: Effects on Growth, Intestinal Immunity, Physical Barrier Function, and Intestinal Microflora. *Frontiers in Immunology*.

[52] Liu S., Xiao G., Wang Q. (2023). Effects of Dietary *Astragalus membranaceus* and *Codonopsis pilosula* Extracts on Growth Performance, Antioxidant Capacity, Immune Status, and Intestinal Health in Broilers. *Frontiers in Veterinary Science*.

[53] He Y., Wang D., Liu K., Deng S., Liu Y. (2023). Sodium Humate Alleviates LPS-Induced Intestinal Barrier Injury by Improving Intestinal Immune Function and Regulating Gut Microbiota. *Molecular Immunology*.

[54] Wang D., He Y., Liu K., Deng S., Fan Y., Liu Y. (2022). Sodium Humate Alleviates Enterotoxigenic *Escherichia coli*-Induced Intestinal Dysfunction via Alteration of Intestinal Microbiota and Metabolites in Mice. *Frontiers in Microbiology*.

[55] Jooné G. K., van Rensburg C. E. J. (2004). An in Vitro Investigation of the Anti-Inflammatory Properties of Potassium Humate. *Inflammation*.

[56] Mudroňová D., Karaffová V., Pešulová T. (2020). The Effect of Humic Substances on Gut Microbiota and Immune Response of Broilers. *Food and Agricultural Immunology*.

[57] Zhou H., Liu Y., Meng X. (2024). Effects of Dietary Pectin on the Growth Performance, Intestinal Barrier, and Antioxidant Status of Juvenile Rainbow Trout (*Oncorhynchus mykiss*). *Aquaculture Reports*.

[58] Tarnecki A. M., Burgos F. A., Ray C. L., Arias C. R. (2017). Fish Intestinal Microbiome: Diversity and Symbiosis Unravelled by Metagenomics. *Journal of Applied Microbiology*.

[59] Xia Y., Lu M., Chen G. (2018). Effects of Dietary, *Lactobacillus rhamnosus*, JCM1136 and *Lactococcus lactis* subsp. Lactis JCM5805 on the Growth, Intestinal Microbiota, Morphology, Immune Response and Disease Resistance of Juvenile Nile Tilapia, *Oreochromis niloticus*. *Fish & Shellfish Immunology*.

[60] Zheng Y., Wu W., Hu G. (2018). Gut Microbiota Analysis of Juvenile Genetically Improved Farmed Tilapia (*Oreochromis niloticus*) by Dietary Supplementation of Different Resveratrol Concentrations. *Fish and Shellfish Immunology*.

[61] Zhang Y., Sun M., Liu Y. (2023). Gut Microbiota Adaptation to Low and High Carbohydrate-to-Protein Ratio Diets in Grass Carp (*Ctenopharyngodon idella*). *Aquaculture Reports*.

[62] Tian J. J., Jin Y. Q., Yu E. M. (2022). Intestinal Farnesoid X Receptor Mediates the Effect of Dietary Berberine on Lipid Accumulation in Grass Carp (*Ctenopharyngodon idella*). *Aquaculture*.

[63] Xue Y. H., Sun Z. X., Xu Z. Y. (2021). Effects of Polyethylene Microplastics Exposure on Intestinal Flora of Zebrafish. *Polish Journal of Environmental Studies*.

[64] Xu Y., Zhu Y., Li X., Sun B. (2020). Dynamic Balancing of Intestinal Short-Chain Fatty Acids: The Crucial Role of Bacterial Metabolism. *Trends in Food Science & Technology*.

[65] Koh A., De Vadder F., Kovatcheva-Datchary P., Bäckhed F. (2016). From Dietary Fiber to Host Physiology: Short-Chain Fatty Acids as Key Bacterial Metabolites. *Cell*.

[66] Li C., Li X., Li P. (2022). Sodium Humate Alters the Intestinal Microbiome, Short-Chain Fatty Acids, Eggshell Ultrastructure, and Egg Performance of Old Laying Hens. *Frontiers in Veterinary Science*.

[67] Vitorino F. G., Hilario A. R., Alves A. L. (2017). The Microbiome of a Striped Dolphin (*Stenella coeruleoalba*) Stranded in Portugal. *Research in Microbiology*.

[68] Tsuchiya C., Sakata T., Sugita H. (2008). Novel Ecological Niche of *Cetobacterium somerae*, An Anaerobic Bacterium in the Intestinal Tracts of Freshwater Fish. *Letters in Applied Microbiology*.

[69] Zhang Z., Fan Z., Yi M. (2022). Characterization of the Core Gut Microbiota of Nile Tilapia (*Oreochromis niloticus*): Indication of a Putative Novel *Cetobacterium* Species and Analysis of Its Potential Function on Nutrition. *Archives of Microbiology*.

[70] Nikolaev Y., Demkina E., Borzenkov I. (2020). Role of the Structure of Humic Substances in Increasing Bacterial Survival. *Open Access Journal of Microbiology & Biotechnology*.

