# Supplementary material for: Dietary Sodium Humate Improves Intestinal Mucosal Immune and Biological Barriers of Genetically Improved Farmed Tilapia (Oreochromis niloticus)
Source: Aquac Nutr. 2025 Oct 9;2025:3398904. doi: 10.1155/anu/3398904 (PMC12530943; doi:10.1155/anu/3398904)
Supplement: Supporting Information — Due to space constraints, formulation and proximate composition of the experimental diets (Table S1) are included in the Supporting Information. [file 3398904.f1.docx]

**Table S1.** Formulation and proximate composition of the experimental diets (%, dry matter basis).

| *Ingredients* | SH0 | SH1 | SH2 | SH4 | SH6 |
| --- | --- | --- | --- | --- | --- |
| Fish meal | 5.00 | 5.00 | 5.00 | 5.00 | 5.00 |
| Soybean meal | 16.30 | 16.30 | 16.30 | 16.30 | 16.30 |
| Rapeseed meal | 11.00 | 11.00 | 11.00 | 11.00 | 11.00 |
| Cottonseed meal | 9.00 | 9.00 | 9.00 | 9.00 | 9.00 |
| Wheat flour | 26.10 | 26.00 | 25.90 | 25.70 | 25.50 |
| Wheat bran | 27.00 | 27.00 | 27.00 | 27.00 | 27.00 |
| Soybean oil | 2.80 | 2.80 | 2.80 | 2.80 | 2.80 |
| Soybean lecithin | 0.50 | 0.50 | 0.50 | 0.50 | 0.50 |
| Ca(H_2_PO_4_)_2_ | 0.80 | 0.80 | 0.80 | 0.80 | 0.80 |
| Choline chloride (50%) | 0.30 | 0.30 | 0.30 | 0.30 | 0.30 |
| Sodium chloride | 0.20 | 0.20 | 0.20 | 0.20 | 0.20 |
| Sodium humate^a^ | 0.00 | 0.10 | 0.20 | 0.40 | 0.60 |
| Mineral mix | 0.50 | 0.50 | 0.50 | 0.50 | 0.50 |
| Vitamin mix | 0.50 | 0.50 | 0.50 | 0.50 | 0.50 |
| *Proximate composition* | | | | | |
| Dry matter (DM, %) | 92.31 | 90.47 | 90.65 | 90.18 | 92.08 |
| Crude protein (% DM) | 29.32 | 29.68 | 29.01 | 29.17 | 29.14 |
| Crude lipid (% DM) | 6.91 | 6.88 | 7.02 | 6.96 | 6.94 |
| Ash (% DM) | 7.00 | 7.10 | 7.04 | 7.14 | 7.15 |
| Gross energy (kJ/g DM) | 20.53 | 20.51 | 20.56 | 20.43 | 20.52 |

^a^ Provided by Beijing Sloan Biological Technology Co., Ltd. (Beijing, China).

^b^ Mineral mix (g/kg mixture): MgSO_4_⋅7H_2_O, 180; KI, 1; FeSO_4_⋅H_2_O, 260; ZnSO_4_⋅H_2_O, 180; GuSO_4_⋅5H_2_O, 25; Na_2_Se_2_O_3_, 0.01; MnSO_4_⋅H_2_O, 180; CoCl_2_⋅6H_2_O, 0.75.

^c^ Vitamin mix (g/kg mixture): retinyl acetate (2,800,000 IU/g), 2; cholecalciferol, 0.03; DL-α-tocopheryl acetate, 30; menadione, 3; thiamine hydrochloride, 8; riboflavin, 11; pyridoxine hydrochloride, 8; vitamin B_12_, 0.02; ascorbic acid, 50; folic acid, 1; biotin, 0.1; niacin, 30; calcium D-pentothenate, 32; inositol, 25.
